# KSIMC: Predicting Kinase–Substrate Interactions Based on Matrix Completion

**DOI:** 10.3390/ijms20020302

**Published:** 2019-01-14

**Authors:** Jingzhong Gan, Jie Qiu, Canshang Deng, Wei Lan, Qingfeng Chen, Yanling Hu

**Affiliations:** 1 School of Computer Science and Engineering, Yulin Normal University, Yulin 537000, China; jgxygjz@126.com (J.G.); jgxyqj@126.com (J.Q.); 2 School of Computer, Electronics and Information, Guangxi University, Nanning 530004, China; dcs288@mail.gxu.cn; 3 State Key Laboratory for Conservation and Utilization of Subtropical Agro-Bioresources, Guangxi University, Nanning 530004, China; 4 Center for Genomic and Personalized Medicine, Guangxi Medical University, Nanning 530021, China; ylhupost@163.com

**Keywords:** protein phosphorylation, kinase-substrate interaction, heterogeneous network, matrix completion

## Abstract

Protein phosphorylation is an important chemical modification catalyzed by kinases. It plays important roles in many cellular processes. Predicting kinase–substrate interactions is vital to understanding the mechanism of many diseases. Many computational methods have been proposed to identify kinase–substrate interactions. However, the prediction accuracy still needs to be improved. Therefore, it is necessary to develop an efficient computational method to predict kinase–substrate interactions. In this paper, we propose a novel computational approach, KSIMC, to identify kinase–substrate interactions based on matrix completion. Firstly, the kinase similarity and substrate similarity are calculated by aligning sequence of kinase–kinase and substrate–substrate, respectively. Then, the original association network is adjusted based on the similarities. Finally, the matrix completion is used to predict potential kinase–substrate interactions. The experiment results show that our method outperforms other state-of-the-art algorithms in performance. Furthermore, the relevant databases and scientific literature verify the effectiveness of our algorithm for new kinase–substrate interaction identification.

## 1. Introduction

Protein phosphorylation is one of the most important post-translational modifications (PSMs) in an organism [1]. It is catalyzed by protein kinases, which promote the transfer of a phosphate group to corresponding substrates. Additionally, protein phosphatases remove the phosphates from substrates. Therefore, protein phosphorylation is a reversible post-translational modification based on the equilibrium of kinases and phosphatases. It plays critical roles in many cellular processes, such as cell metabolism, cell proliferation, cell differentiation, cell apoptosis, and cellular signal transduction [2,3]. Abnormal action of kinases and substrates may lead to a series of diseases, such as rheumatoid arthritis [4] and diabetes [5]. Thus, identifying interactions between substrates and its specific kinases may facilitate the study of diseases and drug targets [6,7,8].

In recent years, several biological methods have been proposed to identify phosphorylation sites and corresponding kinases including a low-throughput [9] and high-throughput [10] technique. Large amounts of phosphorylation sites have been identified by using high-throughput technology. However, most of the corresponding kinases are still unknown. For example, there are more than 30,000 phosphorylation sites stored in the popular knowledgebase Phospho.ELM [11]. However, 90% of these phosphorylation sites do not have records of corresponding annotated kinases. Moreover, a similar problem also exists in the PhosphoSitePlus [12], more than 95% phosphorylation sites do not have the records of the corresponding annotated kinases. Therefore, many computational methods have been developed for identifying kinase–substrate interactions [13,14]. Linding et al. [15] proposed a computational framework to identify a site-specific kinase–substrate based on the network context of kinases and phosphoproteins. Dang et al. [16] developed a new method for identifying kinase–substrate interactions by using conditional random fields. Zhou et al. [17] proposed a web server tool (GPS) to predict kinase–substrate interactions based on the BLOSUM matrix and Markov Cluster Algorithm. Zou et al. [18] presented a computational method, PKIS, to identify kinase–substrate interaction by applying the composition of a monomer spectrum encoding strategy(CMS) [19] to encode the protein sequence feature. Patrick et al. [20] proposed a Bayesian network model to identify kinase–substrate interactions by integrating the cellular context. Fan et al. [21] developed a random forest model for predicting kinase–substrate interactions based on functional information. Li et al. [22] proposed a kernel-based method to identify kinase–substrate interactions by using Supervised Laplacian Regularized Least Squares. Song et al. [23] presented a computational method to infer the relationships between kinases and substrates by integrating protein sequence and functional features. Gnad et al. [24] utilized support vector machines to predict phosphorylation and acetylation sites based on the primary sequence and developed an online database (PHOSIDA) to store phosphorylation data. Moreover, several computational methods employ the biological network information to improve the prediction accuracy. For instance, Song et al. [25] proposed a computational method named iGPS, to predict kinase–substrate interactions based on the PPI network. Damle et al. [26] presented an algorithm, PhosNetConstruct, to infer kinase-substrate interactions based on the domain-specific phosphorylation network. Li et al. [27] proposed a network-based method to identify kinase–substrate interactions by integrating sequence similarity. Qin et al. [28] developed a computational framework for predicting kinase–substrate interactions based on the protein domains network. However, due to the complexity of protein phosphorylation, the accuracy of kinase–substrate prediction of most of the exiting computational methods still needs to be improved.

In this paper, we propose a new computational approach, KSIMC, to predict kinase–substrate interactions based on matrix completion. Firstly, the kinase–kinase similarity and the substrate-substrate similarity are calculated by using a sequence local alignment method, respectively. Then, the kinase–substrate association network is adjusted based on pairwise similarities. Finally, the matrix completion is used to predict potential kinase–substrate interactions. The experiment results show that our method outperforms other state-of-the-art algorithms in performance. Furthermore, the relevant databases and scientific literatures verify the effectiveness of this algorithm for potential kinase–substrate interactions prediction.

## 2. Experiments and Results

### 2.1. Evaluation Metrics

In this paper, ten-fold cross-validation and de novo tests are conducted to evaluate the performance of KSIMC in predicting kinase–substrate interactions. In the ten-fold cross validation, all known kinase–substrate associations are randomly divided into 10 subsets of equal size. Each subset takes a turn as a test set, while the remaining nine subsets are treated as the training set. After performing the algorithm on the dataset, the predicted scoring matrix of all kinase–substrate interactions is generated. Then we calculate the true positive (*TP*), true negative (*TN*), false positive (*FP*) and false negative (*FN*) by ranking the prediction results. Correspondingly, *TP* represents the number of the positive samples that are correctly predicted, *TN* represents the number of the negative samples that are correctly predicted, *FP* represents the number of the positive samples that are incorrectly predicted, and *FN* represents the number of the negative samples that are incorrectly predicted. By changing various thresholds, true positive rate (*TPR*) and false positive rate (*FPR*) are calculated as follows:

(1) $$TPR=\frac{TP}{TP+FN}$$

(2) $$FPR=\frac{FP}{FP+TN}$$

Finally, the Receiver Operating Characteristic (*ROC*) is drawn based on the *TPR* and *FPR* and the Area Under Curve (*AUC*) is calculated for the performance evaluation.

### 2.2. Comparison with Network-Based Method

**Figure 1:**
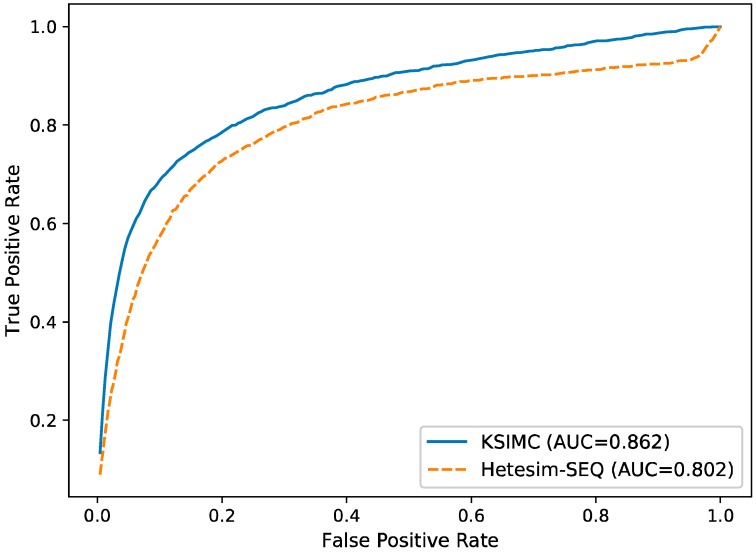
The ROC curves for predicting kinase–substrate interactions with different methods.

To evaluate the performance of KSIMC, we compare it with another network-based method Hetesim-SEQ [27] for all kinases and substrates by using ten-fold cross-validation. Hetesim-SEQ is a network-based method for kinase–substrate interactions prediction based on Hetesim [29] similarity. The ROC curve of KSIMC and Hetesim-SEQ is shown in Figure 1. KSIMC achieves the AUC value of 0.862, which is 0.06 higher than Hetesim-SEQ. It shows that KSIMC performs better than Hetesim-SEQ.

### 2.3. Comparison with Different Predictors by De Novo Test

**Figure 2:**
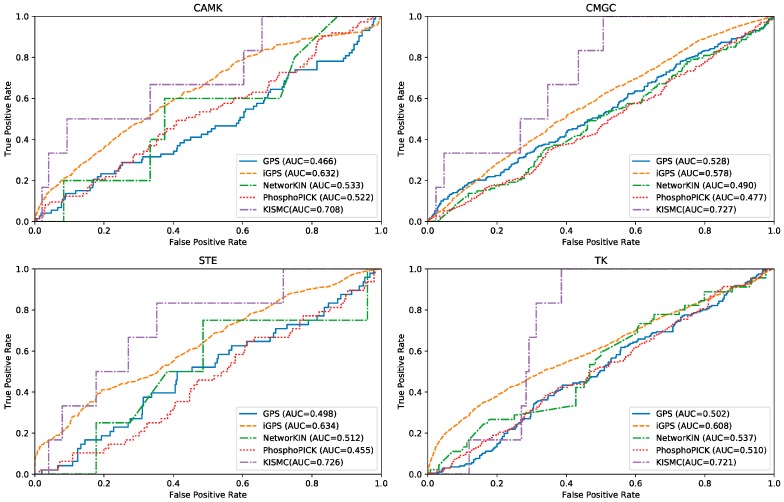
The ROC curves for kinase group CAMK, CMGC, STE and TK with different algorithms.

In order to evaluate the ability of KSIMC in predicting potential kinase–substrate interactions, we perform de novo kinase–substrate interaction prediction test experiments. In the de novo test, for each queried kinase *i*, all known kinase–substrate interactions of kinase *i* are deleted. The remaining kinase–substrate interactions are treated as training sets. Four popular methods of kinase–substrate interactions including GPS [17], iGPS [25], NetworKIN [15] and PhosphoPICK [20] are also applied to predict potential substrates for new kinases. Since these predictors only provide a web server, we submit the dataset to the corresponding web server for testing. Four kinase groups including CAMK, CMGC, STE, and TK are used to illustrate the overall performance of different methods. The ROC curves for different methods in different kinase groups are illustrated in Figure 2. It can be observed that KSIMC performs better than the other four algorithms on different kinase groups. For instance, for the CAMK kinase group, the AUC value of KSIMC is 0.813, which is 0.242, 0.076, 0.175 and 0.186 higher than GPS, iGPS, NetworKIN and PhosphoPICK, respectively. Similarly, for the CMGC kinase group, the AUC value of KSIMC is 0.199, 0.149, 0.237, and 0.25 higher than GPS, iGPS, NetworKIN and PhosphoPICK, respectively.

### 2.4. Case Studies

To further demonstrate the ability of KSIMC to predict new kinase–substrate interactions, the case study is performed in here. All known kinase–substrate interactions are treated as the training set and the unknown kinase–substrate interactions are treated as the test set. We apply KSIMC to predict potential kinase–substrate interactions and obtain the prediction scores for all candidate kinase–substrate interactions (Table S1). We take the substrate IRS1 as an example to illustrate the capability of KSIMC to identify unknown kinase–substrate interactions. The top 10 predicted results of IRS1 are selected to validate based on the database and literatures (Table S2). The detailed information is shown in Table 1. We find that four predicted kinases have been confirmed in the PhosphoNET database and one predicted kinase has been validated in recent literature. For example, the serine site at the 312 position of the IRS1 sequence is catalyzed by two kinases (MAPK1 ranked at top 2 and MAPK8 ranked at top 6). The serine sites at the position 24 and 233 of IRS1 are catalyzed by PRKCA (ranked at top 3) and PRKCE (ranked at top 7), respectively. In addition, it has been proved that IRS1 can be regulated by CDK1 [30].

In addition, some interesting kinases such as ABL1, CSNK2A1, GSK3B, PRKG1 and RPS6KA3 are also discovered from the experimental results. The molecular mechanism of these kinases is still unknown; it deserves a biologist to validate its functions by using a biological experiment.

## 3. Materials and Methods

### 3.1. Data Resources

In this work, the human kinase–substrate interactions are obtained from the Phospho.ELM 9.0 database [11]. The interactions labeled with kinase group or family are not considered in the experiment. After removing the redundant data, 216 kinases, 724 substrates and 1256 kinase-substrate interactions are collected in final. Many kinases (substrates) are only related with individual substrate(kinases). There are 78 kinases with only one related substrate and 454 substrates with only one related kinase. The corresponding protein sequence data of 216 kinase and 724 substrates are downloaded from the UniProt (http://www.uniprot.org/) (10/02/2018) database.

### 3.2. Kinase-Kinase and Substrate-Substrate Similarity Measure

Based on the protein sequence information of the kinase and the substrate, the sequence local alignment method is used to calculate the kinase–kinase similarity and the substrate–substrate similarity. The sequence alignment tool Emboss [31] is utilized to calculate the sequence similarity, which has been widely used in sequence alignment [32,33]. The parameters of Emboss are set with the default value (Matrix = BLOSUM62, Gap open = 10, Gap extend = 0.5).

### 3.3. Adjust the Kinase-Substrate Interaction Network

**Figure 3:**
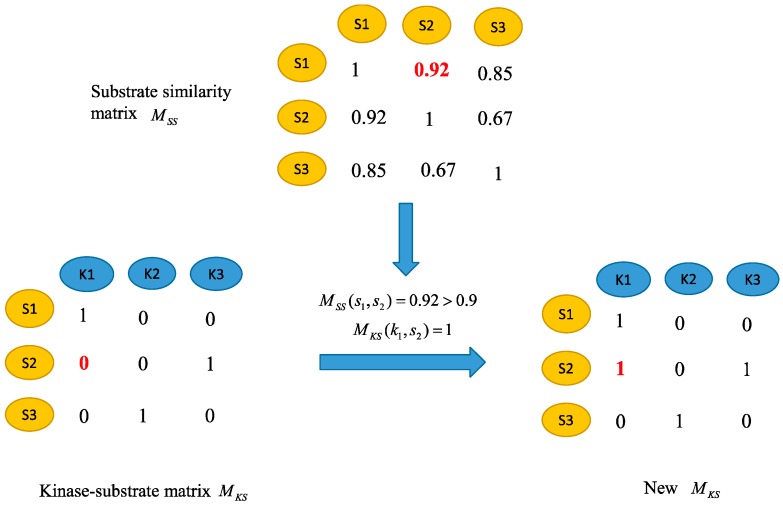
Adjust the kinase–substrate association network.

The adjacency matrix of the kinase–substrate interaction network is described as *M_KS_* matrix. If there is a known relationship between the kinase *i* and the substrate *j*, then *M_KS_*(*i*,*j*) is 1, otherwise *M_KS_*(*i*,*j*) is 0. However, there may still be potential positives for unknown kinase-substrate relationships. In order to further enhance the reliability of the kinase–substrate association network, the kinase–substrate association network is adjusted based on sequence similarity. It is based on the assumption that similar substrates tend to be related with similar kinases. For instance, assume that kinase *k*_1_ is associated with the substrate *s*_1_ and there is no known association between *k*_1_ and the substrate *s*_2_. If the similarity between *s*_1_ and *s*_2_ is greater than a certain threshold *t*, the kinase *k*_1_ and substrate *s*_2_ are considered to be associated and the corresponding element in the kinase–substrate interaction matrix is set to 1. The parameter *t* is set as 0.9 here. Based on the above process, the kinases–substrate associations are readjusted to obtain a new kinase–substrate interaction matrix. The process is shown in Figure 3.

### 3.4. Construction of Kinase-Substrate Heterogenous Network

**Figure 4:**
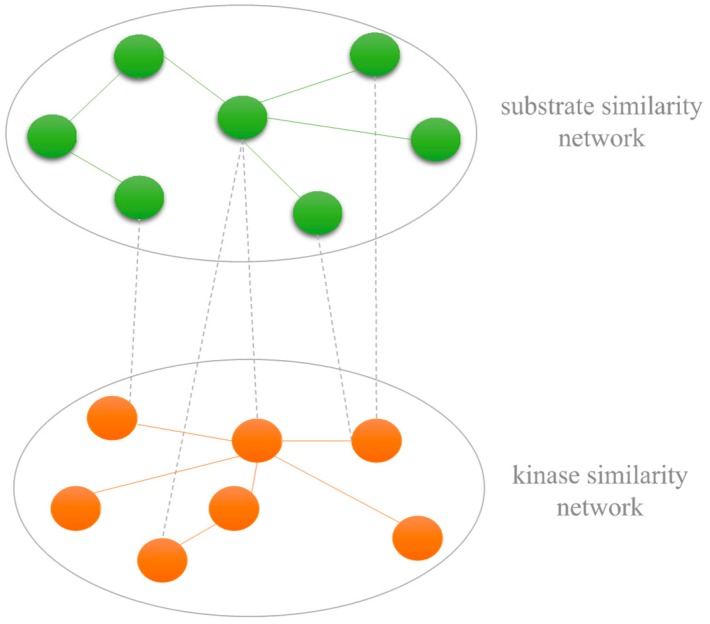
An example of the kinase–substrate heterogenous network.

The heterogeneous network is constructed based on three sub-networks including the kinase similarity network, substrate similarity network and kinase–substrate association network. Let $K=\left\{K_{1},K_{2},\cdots ,K_{m}\right\}$ represent the set of *m* different kinases. *M_kk_* denotes the kinase similarity matrix. Similarly, for the substrate similarity network, let $S=\left\{S_{1},S_{2},\cdots ,S_{n}\right\}$ represent the set of *n* different substrates. *M_ss_* denotes the substrate similarity matrix. The value of element *M_ss_* denotes the similarity of two substrates. The example of the kinase–substrate heterogenous network is shown in Figure 4.

The matrix *M* of the kinase–substrate heterogenous network can be defined as follows:

(3) $$M=\left[\begin{array}{cc} M_{KK} & M_{KS}\\ M_{KS}^{T} & M_{SS} \end{array}\right]$$

The main diagonal elements of matrix *M* are composed of sub-matrices $M_{KK}$ and $M_{SS}$. The sub-diagonal elements are composed of sub-matrices $M_{KS}$ and $M_{KS}^{T}$. $M_{KS}^{T}$ is the transpose of $M_{KS}$.

### 3.5. Predicting Kinase-Substrate Interactions by Using Matrix Completion

The goal of the kinase–substrate interactions prediction is to complement the heterogeneous network adjacency matrix *M* by constructing a matrix M*. The adjacency matrix *M* can be recovered by minimizing the rank of the matrix based on the assumption that the matrix is of low rank. The optimization problem can be defined as:

(4) $$\min\left(rank\left(M^{*}\right)\right)\;s.t.P_{\Omega }\left(M^{*}\right)=P_{\Omega }\left(M\right)$$

where M* is a candidate solution matrix with scores of all the unknown kinase–substrate interactions. Ω denotes a set of index of known elements in the matrix *M*. $P_{\Omega }$ denotes an orthogonal projection matrix. It is defined as follows:

(5) $$P_{\Omega }\left(X\right)=\{\begin{array}{c} X,\left(i,j\right)\in \Omega \\ 0,\left(i,j\right)\notin \Omega \end{array}$$

However, the rank minimization problem is known to be NP-Hard [34]. It is impractical for the problem of predicting kinase–substrate interactions with a large number of kinases and substrates. In order to facilitate the solution, the relaxation form [35] is used to minimize the nuclear norm with a soft threshold instead of minimizing the rank:

(6) $$\min\left(\tau \|M^{*}\|+\frac{1}{2}\|M^{*}{\|}_{F}^{2}\right)\;s.t.P_{\Omega }\left(M^{*}\right)=P_{\Omega }\left(M\right)$$

where $\left\|M^{*}\right\|$ denotes the nuclear norm of the matrix M*. $\|M^{*}{\|}_{F}$ denotes the Frobernius form of M* and τ is a singular value threshold parameter.

**Figure 5:**
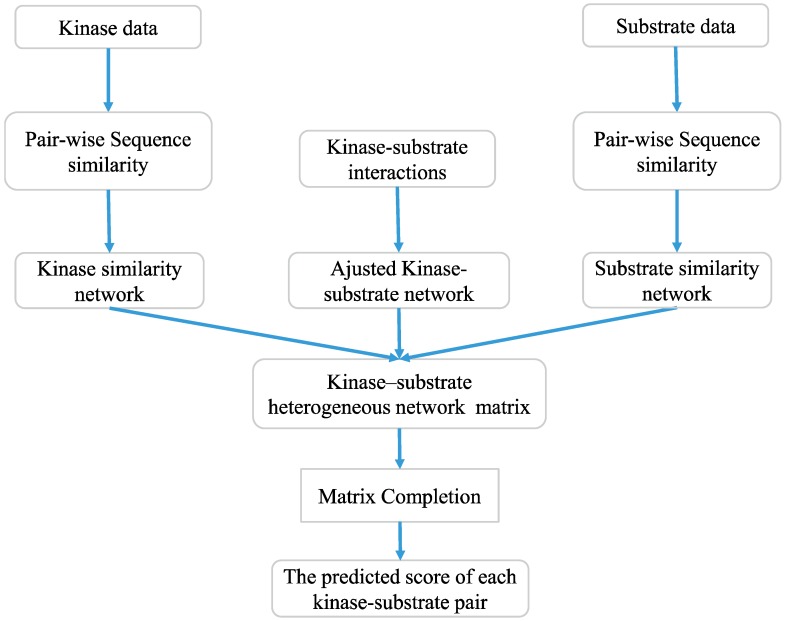
The flow chart of potential kinase–substrate interactions identification by using matrix completion.

This optimization problem can be solved by the singular value thresholding (SVT) algorithm [35]. For a matrix X with rank *r*, singular value decomposition of $X\in R^{n1\times n2}$ is as follows:

(7) $$X=U\sum V^{*},\sum =\mathrm{diag}\left({\left\{\sigma_{i}\right\}}_{1\leq i\leq r}\right)$$

where *U* and *V* are n1×r and r×n2, respectively. $\sigma_{i}$ is a nonnegative singular value. For each τ>0, the soft thresholding operator $D_{\tau }$ is defined as follows:

(8) $$D_{\tau }\left(X\right)={\mathrm{UD}}_{\tau }\left(\sum \right)V^{*},D_{\tau }\left(\sum \right)=\mathrm{diag}\left({\left(\sigma_{i}-\tau \right)}_{+}\right)$$

where ${\left(\sigma_{i}-\tau \right)}_{+}$ denotes the positive part of $\left(\sigma_{i}-\tau \right)$, namely ${\left(\sigma_{i}-\tau \right)}_{+}=\max\left(0,\left(S_{i}-t\right)\right)$. This soft thresholding operation thus shrinks the singular values of *X* toward zero. The shrinkage iterations starts from *Y*^0^, and the matrix *X^K^* and *Y^K^* are reconstructed continuously via:

(9) $$\{\begin{array}{c} X^{K}=D_{\tau }\left(Y^{K-1}\right)\\ Y^{K}=Y^{K-1}+\delta P_{\Omega }\left(M-X^{K}\right) \end{array}$$

where δ is the positive step size. The value of δ is set to $\left(m+n\right)/\sqrt{\left|\Omega \right|}$, and *Y*^0^ is set to $\lceil \tau /(\delta \|P_{\Omega }\left(M\right)\|)\rceil \delta P_{\Omega }\left(M\right)$ as suggested by previous research [36]. Since the algorithm needs to iteratively decompose and reconstruct the matrix, a fast implementation of the SVT algorithm [37] is used to improve the computational efficiency. The flow chart of predicting kinase–substrate interactions by using matrix completion is shown in Figure 5.

## 4. Conclusions

Protein phosphorylation is one of the most post-translational modifications, which plays critical roles in many cellular processes, such as cell metabolism, gene expression and cellular signal transduction. Abnormal action of kinases and substrates may lead to a series of diseases. Thus, identification of the interactions between substrates and its specific kinases can increase our understanding in the pathogenesis of diseases [38,39,40]. In this paper, we propose a computational method to predict kinase-substrate interactions by using matrix completion. Firstly, the kinase similarity matrix and the substrate similarity matrix are calculated by aligning the sequence of kinase–kinase and substrate–substrate, respectively. Then the original association network is adjusted and the adjacency matrix of the kinase–substrate heterogeneous network is constructed based on the similarities. Finally, the matrix completion is used to fill in the missing information in the adjacency matrix and to predict the potential kinase–substrate interactions. The experiment results show that our method outperforms other state-of-the-art algorithms in performance. In addition, as KSIMC only utilizes the sequence information, it also can be used to predict the kinase–substrate of other species such as prokaryotes.

## Figures and Tables

**Table 1:** The top 10 potential kinases of IRS1 predicted by KSIMC.

Top	Substrate	Predicted Kinase	Evidence
1	IRS1	CDK1	PMID: 20798132
2	IRS1	MAPK1	PhosphoNET
3	IRS1	PRKCA	PhosphoNET
4	IRS1	ABL1	Unknown
5	IRS1	CSNK2A1	Unknown
6	IRS1	MAPK8	PhosphoNET
7	IRS1	PRKCE	PhosphoNET
8	IRS1	GSK3B	Unknown
9	IRS1	PRKG1	Unknown
10	IRS1	RPS6KA3	Unknown

## Supplementary Materials

Supplementary materials can be found at http://www.mdpi.com/1422-0067/20/2/302/s1.

## Abbreviations

KSIMC	Predicting kinase-substrate interactions based on matrix completion
AUC	Area Under roc Curve
ROC	Receiver Operating Characteristic Curve
TP	True Positive
FP	False Positive
TN	True Negative
FN	False Negative
TPR	True Positive Rate
FPR	False Positive Rate
