# Supplementary material for: KSIMC: Predicting Kinase–Substrate Interactions Based on Matrix Completion
Source: Int J Mol Sci. 2019 Jan 14;20(2):302. doi: 10.3390/ijms20020302 (PMC6358935; doi:10.3390/ijms20020302)
Supplement: Supplementary file 1 [file ijms-20-00302-s001.pdf]

Table S1. The top-10 potential kinases of SGK1 predicted by KSIMC.

top	Substrate	Predicted kinase	Evidence
1	SGK1	PLK3	PMID: 19619128
2	SGK1	MELK	Unknown
3	SGK1	PTK2B	Unknown
4	SGK1	PAK1	PMID: 26219339
5	SGK1	RIPK4	Unknown
6	SGK1	STK11	PMID: 26827666
7	SGK1	TESK1	Unknown
8	SGK1	LCK	Unknown
9	SGK1	PRKG2	Unknown
10	SGK1	GRK2	PMID: 26013542

The table S1 lists the top-10 predicted results of SGK1 are selected to validate based on the literatures. We find that four predicted kinases have been confirmed in recent literature. For example, it has been proved that PLK3 can regulate the SGK1.

Table S2. The top-10 potential kinases of SNAP23 predicted by KSIMC.

top	Substrate	Predicted kinase	Evidence
1	SNAP23	SRC	PMID: 20888376
2	SNAP23	FGFR1	Unknown
3	SNAP23	STK10	Unknown
4	SNAP23	PRKCB	Unknown
5	SNAP23	RIPK4	Unknown
6	SNAP23	CHEK2	Unknown
7	SNAP23	GRK2	PMID: 24904059
8	SNAP23	CDK2	PMID: 12877659
9	SNAP23	INSR	Unknown
10	SNAP23	MAP3K1	Unknown

The table S2 lists the top-10 predicted results of SNAP23 are selected to validate based on the literatures. We find that three predicted kinases have been confirmed in recent literature. For example, it has been discovered that SNAP23 is required for integrin signaling through SRC-dependent pathway.

Kinase-substrate interaction data

O00238	Q15796
O00238	Q15797
O00329	O00329
O14733	P45983
O14733	P45984
O14733	P53779
O14757	O15350
O14757	P04637
O14757	P30304
O14757	P30305
O14757	P33991
O14757	Q9UKI8
O14920	O43524
O14920	P19838
O14920	Q04206
O14920	Q9Y6K9
O14965	O14965
O14965	O95983
O14965	P04637
O14965	P30305
O14965	P38398
O14965	P49450
O14965	P68431
O14965	Q9NRM7
O15111	P19838
O15111	P24385
O15111	Q00653
O15111	Q04206
O15111	Q9Y618
O15264	O00418
O15264	P16949
O43293	O43293
O43293	P24844
O43293	P40429
O43293	P68431
O43293	Q00987
O43293	Q96A00
O43318	O43318
O43781	P16220
O60285	P55212

O60674	O60674
O60674	P10912
O60674	P16410
O60674	P19235
O60674	P40763
O60674	P41597
O60674	P42224
O60674	P42229
O60674	Q9NRF2
O75116	Q16555
O75582	O75582
O75582	P0C0S8
O75582	Q04206
O75914	O75914
O75914	P04049
O75914	P19429
O76003	O43516
O76003	P32942
O76003	P35568
O76003	Q04759
O76003	Q8IV61
O76003	Q9UEW8
O94804	P26038
O94804	P53350
O96017	O96017
O96017	P04637
O96017	P30304
O96017	P30307
O96017	P38398
O96017	Q01094
P00519	O15162
P00519	O15350
P00519	O43586
P00519	P00519
P00519	P04040
P00519	P04083
P00519	P07203
P00519	P11274
P00519	P11387
P00519	P15941
P00519	P25963

P00519	P29350
P00519	P43351
P00519	P46108
P00519	P46109
P00519	P55211
P00519	Q00535
P00519	Q00987
P00519	Q06187
P00519	Q06609
P00519	Q15139
P00519	Q99638
P00519	Q9NRQ2
P00519	Q9Y6W5
P00533	O14964
P00533	O15492
P00533	P00533
P00533	P04083
P00533	P11171
P00533	P15311
P00533	P15941
P00533	P18031
P00533	P19174
P00533	P20936
P00533	P24844
P00533	P51692
P00533	P52735
P00533	P61925
P00533	P62158
P00533	P62993
P00533	Q13480
P04049	Q02750
P04626	P04626
P04629	P04629
P05129	Q03721
P05129	Q9UBS0
P05771	P05107
P05771	P11388
P05771	P15336
P05771	P20700
P05771	P35637
P05771	Q03721

P05771	Q9UBS0
P06213	P06213
P06213	P18031
P06213	P22681
P06213	P27986
P06213	P53004
P06213	P62158
P06213	Q13480
P06213	Q99704
P06239	O14543
P06239	P03372
P06239	P06127
P06239	P06239
P06239	P10747
P06239	P14784
P06239	P15311
P06239	P15498
P06239	P16885
P06239	P20138
P06239	P25963
P06239	P29350
P06239	P43403
P06239	P52735
P06239	P60484
P06239	Q04759
P06239	Q08881
P06239	Q13094
P06239	Q13291
P06239	Q13813
P06239	Q96LC7
P06239	Q9UN19
P06239	Q9UQQ2
P06241	O15117
P06241	O43294
P06241	P06241
P06241	P11137
P06241	P11912
P06241	P12318
P06241	P16885
P06241	P20963
P06241	P22681

P06241	P30419
P06241	P35222
P06241	P37840
P06241	P40259
P06241	Q03135
P06241	Q13291
P06241	Q13322
P06241	Q14643
P06241	Q86WV1
P06241	Q96J02
P06493	O00443
P06493	O00562
P06493	O00571
P06493	O14757
P06493	O14994
P06493	O15287
P06493	O15392
P06493	O95835
P06493	O95997
P06493	P00533
P06493	P04183
P06493	P05787
P06493	P06748
P06493	P08670
P06493	P11388
P06493	P12272
P06493	P15172
P06493	P15927
P06493	P17096
P06493	P17706
P06493	P18031
P06493	P22314
P06493	P23443
P06493	P27816
P06493	P30260
P06493	P30291
P06493	P30304
P06493	P30305
P06493	P30307
P06493	P31350
P06493	P36956

P06493	P39748
P06493	P39880
P06493	P46060
P06493	P47736
P06493	P48200
P06493	P49459
P06493	P52732
P06493	P52926
P06493	P55211
P06493	P61020
P06493	P62136
P06493	P67870
P06493	P68400
P06493	Q01094
P06493	Q02750
P06493	Q06481
P06493	Q06830
P06493	Q07666
P06493	Q08050
P06493	Q08379
P06493	Q13415
P06493	Q14674
P06493	Q14739
P06493	Q14807
P06493	Q15149
P06493	Q53EZ4
P06493	Q8IXJ6
P06493	Q8N122
P06493	Q8TAP9
P06493	Q92574
P06493	Q92993
P06493	Q96KB5
P06493	Q99638
P06493	Q9H1A4
P06493	Q9H8V3
P06493	Q9UJX2
P06493	Q9UNZ2
P06493	Q9Y6I3
P07332	P07332
P07332	P11274
P07333	P07333

P07947	P07947
P07947	P22681
P07948	O15492
P07948	P02730
P07948	P07948
P07948	P11137
P07948	P11912
P07948	P12318
P07948	P14317
P07948	P15391
P07948	P16885
P07948	P19235
P07948	P30419
P07948	P31994
P07948	P31995
P07948	P40259
P07948	P42229
P07948	P68400
P07948	Q06187
P07948	Q12972
P07948	Q13291
P07948	Q9HBA0
P07949	O15530
P07949	P07949
P08069	P07550
P08069	P08069
P08581	P08581
P08581	Q13480
P08631	P08631
P08631	P11274
P08631	P42768
P08631	P51692
P08631	Q13444
P08631	Q13905
P09619	P06241
P09619	P09619
P09619	Q06124
P09769	P09769
P09769	P14317
P09769	P68400
P10275	Q13158

P10721	P10721
P11309	P30304
P11309	P38936
P11362	O60506
P11362	P11362
P11362	P51812
P11362	P56945
P11362	Q14247
P11802	P17480
P11802	P28749
P11802	P84022
P11802	Q08999
P12931	O14746
P12931	O15162
P12931	O15357
P12931	O15492
P12931	O15530
P12931	O43597
P12931	O95297
P12931	P00533
P12931	P01350
P12931	P03372
P12931	P04049
P12931	P04083
P12931	P05106
P12931	P06396
P12931	P06733
P12931	P07355
P12931	P08069
P12931	P10301
P12931	P10398
P12931	P10721
P12931	P12931
P12931	P15311
P12931	P15941
P12931	P16885
P12931	P19022
P12931	P20138
P12931	P22001
P12931	P25963
P12931	P27986

P12931	P29350
P12931	P30411
P12931	P30443
P12931	P35222
P12931	P36544
P12931	P41743
P12931	P42224
P12931	P42229
P12931	P46527
P12931	P49023
P12931	P49407
P12931	P51636
P12931	P51692
P12931	P51813
P12931	P56945
P12931	P60953
P12931	P61978
P12931	P63244
P12931	Q00610
P12931	Q03135
P12931	Q05397
P12931	Q07954
P12931	Q13177
P12931	Q13322
P12931	Q13444
P12931	Q13813
P12931	Q13905
P12931	Q14118
P12931	Q14247
P12931	Q15139
P12931	Q92558
P12931	Q99961
P12931	Q9NRY4
P16234	P16234
P17252	O00161
P17252	O14745
P17252	O14939
P17252	P00533
P17252	P00749
P17252	P02786
P17252	P04083

P17252	P05107
P17252	P06127
P17252	P06213
P17252	P07355
P17252	P08567
P17252	P09038
P17252	P09758
P17252	P10721
P17252	P11831
P17252	P13569
P17252	P14598
P17252	P16070
P17252	P16144
P17252	P16150
P17252	P17252
P17252	P18031
P17252	P18206
P17252	P19086
P17252	P19174
P17252	P21399
P17252	P21730
P17252	P25098
P17252	P29350
P17252	P29966
P17252	P30086
P17252	P30411
P17252	P35367
P17252	P35579
P17252	P35611
P17252	P35612
P17252	P35869
P17252	P38936
P17252	P41180
P17252	P42261
P17252	P43119
P17252	P49768
P17252	P49802
P17252	P51674
P17252	P56537
P17252	P60983
P17252	P78314

P17252	Q00169
P17252	Q01954
P17252	Q03721
P17252	Q05209
P17252	Q09472
P17252	Q13224
P17252	Q13507
P17252	Q13769
P17252	Q13976
P17252	Q15080
P17252	Q15139
P17252	Q15762
P17252	Q16760
P17252	Q7KZI7
P17252	Q96A00
P17252	Q99418
P17252	Q9H1D0
P17252	Q9NXH3
P17252	Q9UBS0
P17612	Q13085
P17612	Q9UD71
P17948	P17948
P19525	P05198
P19525	P19525
P21127	O00303
P22455	P22455
P22607	P22607
P23443	P23443
P23443	P23588
P23443	P42345
P23443	P62753
P23443	Q9BY77
P23458	P15260
P23458	P29597
P23458	P42224
P23458	Q9NRF2
P24723	P05107
P24723	Q15139
P24941	O15350
P24941	O75533
P24941	P04183

P24941	P06401
P24941	P10244
P24941	P17480
P24941	P24864
P24941	P33981
P24941	P33991
P24941	P38398
P24941	P38432
P24941	P38936
P24941	P46527
P24941	P49459
P24941	P50613
P24941	P54198
P24941	P84022
P24941	Q02363
P24941	Q02535
P24941	Q08050
P24941	Q08999
P24941	Q8WXE1
P24941	Q99741
P25098	P05387
P25098	P07550
P25098	P09619
P25098	P15311
P25098	P18545
P25098	P21462
P25098	P32245
P25098	P35367
P25098	P37840
P25098	P41143
P25098	Q13509
P27361	O14994
P27361	O43521
P27361	O75582
P27361	P00533
P27361	P01100
P27361	P02686
P27361	P03372
P27361	P05787
P27361	P06239
P27361	P06401

P27361	P08047
P27361	P10242
P27361	P10828
P27361	P11388
P27361	P14598
P27361	P17480
P27361	P17542
P27361	P17655
P27361	P17676
P27361	P19419
P27361	P19793
P27361	P23396
P27361	P24928
P27361	P27708
P27361	P28562
P27361	P35568
P27361	P36956
P27361	P41212
P27361	P42229
P27361	P42702
P27361	P49715
P27361	P49841
P27361	P51812
P27361	P55211
P27361	P56270
P27361	P61978
P27361	P78536
P27361	Q01892
P27361	Q05682
P27361	Q07820
P27361	Q12772
P27361	Q13322
P27361	Q13485
P27361	Q14005
P27361	Q14247
P27361	Q14934
P27361	Q15648
P27361	Q15788
P27361	Q15796
P27361	Q16828
P27361	Q9BUB5

P27361	Q9NQ66
P27361	Q9NRF2
P27361	Q9NYV6
P27361	Q9UJY1
P27361	Q9UQC2
P28482	O00213
P28482	O00562
P28482	O43521
P28482	O75582
P28482	P00533
P28482	P01100
P28482	P01106
P28482	P02686
P28482	P03372
P28482	P06239
P28482	P06401
P28482	P08047
P28482	P10828
P28482	P11388
P28482	P17480
P28482	P17655
P28482	P19793
P28482	P23396
P28482	P24928
P28482	P27708
P28482	P28562
P28482	P36956
P28482	P37231
P28482	P38936
P28482	P42229
P28482	P42702
P28482	P43354
P28482	P46527
P28482	P47712
P28482	P49137
P28482	P49715
P28482	P49795
P28482	P49815
P28482	P49841
P28482	P50548
P28482	P53355

P28482	P55211
P28482	P84022
P28482	Q02750
P28482	Q04206
P28482	Q07820
P28482	Q07889
P28482	Q12772
P28482	Q13153
P28482	Q13322
P28482	Q13480
P28482	Q14005
P28482	Q14674
P28482	Q14934
P28482	Q15648
P28482	Q15788
P28482	Q15797
P28482	Q16828
P28482	Q8IW41
P28482	Q9BY84
P28482	Q9NRF2
P29317	O14493
P29320	P29320
P29322	P29322
P29323	P10301
P29323	P52799
P29597	P17181
P29597	P29597
P29597	P63244
P29597	Q13651
P30291	P06493
P30530	P30530
P31152	P31152
P31751	P03372
P31751	P15311
P31751	P98170
P31751	Q99683
P32298	P30411
P33981	O96017
P33981	P00519
P33981	P04637
P33981	P33981

P33981	P38646
P33981	Q53HL2
P34947	P07550
P34947	P34947
P34947	P37840
P35626	P51681
P35916	P29353
P35916	P35916
P35968	P35916
P35968	P35968
P36507	P27361
P36888	P36888
P36897	Q15796
P37173	P37173
P41240	P06241
P41240	P07948
P41240	P08575
P41240	P09769
P41240	P12931
P41279	P36507
P41279	Q02750
P41743	Q9UBS0
P42336	P09493
P42336	P27986
P42338	P42338
P42345	P23443
P42345	P42345
P42345	Q13541
P42345	Q9UBS0
P42680	P42680
P42680	P51813
P42681	P16410
P42681	P42681
P42681	Q13094
P42684	O15304
P42684	P04040
P42684	P07203
P43250	O14745
P43250	Q15722
P43403	P29353
P43403	P43403

P43403	P51452
P43403	Q13094
P43403	Q16539
P43403	Q9UJU6
P43403	Q9UQC2
P43403	Q9UQQ2
P43405	O43914
P43405	P02730
P43405	P05106
P43405	P12318
P43405	P14317
P43405	P17252
P43405	P19174
P43405	P22681
P43405	P29353
P43405	P31995
P43405	P37840
P43405	P43405
P43405	P51452
P43405	Q13094
P43405	Q14289
P43405	Q8WV28
P43405	Q92918
P45983	O43521
P45983	P05412
P45983	P05787
P45983	P10415
P45983	P15336
P45983	P31946
P45983	P31947
P45983	P37231
P45983	P54259
P45983	P61978
P45983	P63104
P45983	P98177
P45983	Q06481
P45983	Q07817
P45983	Q9UQF2
P45984	O43521
P45984	P05412
P45984	P98177

P45984	Q12968
P45984	Q8IW41
P45984	Q8WYK2
P45984	Q9NYV6
P45984	Q9UQF2
P45985	P19793
P45985	P45983
P45985	P45984
P45985	P45985
P45985	P53778
P45985	P53779
P46734	P46734
P46734	P53778
P48729	O60260
P48729	P02730
P48729	P07910
P48729	P17931
P48729	P27348
P48729	P37840
P48729	P56817
P48729	P63104
P48729	Q12778
P48729	Q9Y2W7
P48730	P12830
P48730	P49810
P48730	P56817
P48736	P48736
P49137	O00418
P49137	O95816
P49137	P04792
P49137	P07101
P49137	P08670
P49137	P09917
P49137	P11831
P49137	P16220
P49137	P30305
P49137	P33241
P49137	P49137
P49137	P49815
P49137	Q00613
P49674	O15055

P49674	P25054
P49674	P49674
P49759	P18031
P49840	P01106
P49840	P05412
P49840	P24864
P49840	Q9BYG3
P49841	O75030
P49841	O95863
P49841	P01106
P49841	P03372
P49841	P05067
P49841	P10636
P49841	P13807
P49841	P15941
P49841	P19838
P49841	P24864
P49841	P30281
P49841	P38936
P49841	P41236
P49841	P47736
P49841	P49768
P49841	P54252
P49841	P60484
P49841	Q04206
P49841	Q07820
P49841	Q13765
P49841	Q14103
P49841	Q14195
P49841	Q16555
P49841	Q9BYG3
P50613	P03372
P50613	P04637
P50613	P06493
P50613	P10276
P50613	P11802
P50613	P14859
P50613	P50613
P50613	Q01094
P50613	Q14847
P50613	Q9UPZ9

P50750	P24928
P51451	P12318
P51451	P31994
P51617	P40763
P51617	P51617
P51812	P01100
P51812	P16220
P51812	P18848
P51812	P46527
P51812	P53355
P51812	Q06413
P51812	Q14934
P51812	Q92934
P51812	Q9NYV6
P51813	Q96T51
P51955	O14777
P51955	P36873
P52333	P23458
P52333	P52333
P52333	Q96LC7
P52564	P53778
P52564	Q16539
P52564	Q9NQU5
P53350	O60566
P53350	O96017
P53350	P06748
P53350	P13693
P53350	P14635
P53350	P30260
P53350	P30291
P53350	P30307
P53350	P35568
P53350	P51587
P53350	Q13526
P53350	Q99640
P53350	Q9H1A4
P53350	Q9UJX2
P53350	Q9UJX3
P53350	Q9UJX5
P53350	Q9Y266
P53350	Q9Y2I6

P53355	P04637
P53355	P25205
P53355	P53355
P53355	Q14457
P53355	Q96RR4
P53667	P23528
P53667	Q9Y281
P53671	P23528
P53778	P53778
P53778	Q13424
P53779	O43521
P53779	P54259
P53779	P61978
P54753	P54753
P54764	P54764
P57059	P35568
P57078	P05107
P57078	P05787
P57078	P08581
P57078	P15941
P57078	P30086
P57078	P31946
P57078	P35568
P57078	P40189
P57078	P40763
P57078	P48050
P57078	P53671
P57078	P61978
P57078	Q09472
P57078	Q15139
P57078	Q9UBS0
P67870	P67870
P67870	Q96SB4
P68400	O76061
P68400	O95477
P68400	P00736
P68400	P04004
P68400	P04198
P68400	P05412
P68400	P05455
P68400	P06127

P68400	P06401
P68400	P06493
P68400	P07900
P68400	P08238
P68400	P08575
P68400	P09651
P68400	P10242
P68400	P10646
P68400	P11274
P68400	P11388
P68400	P11473
P68400	P11717
P68400	P11831
P68400	P12259
P68400	P15884
P68400	P18887
P68400	P19447
P68400	P25963
P68400	P30305
P68400	P31749
P68400	P32121
P68400	P35638
P68400	P37840
P68400	P42768
P68400	P45984
P68400	P48426
P68400	P49427
P68400	P49810
P68400	P55010
P68400	P60484
P68400	P60983
P68400	P61244
P68400	Q00613
P68400	Q02078
P68400	Q04206
P68400	Q04724
P68400	Q08945
P68400	Q13323
P68400	Q13547
P68400	Q13563
P68400	Q15185

P68400	Q15287
P68400	Q16543
P68400	Q16666
P68400	Q712K3
P68400	Q92769
P68400	Q96SB4
P68400	Q9UBF6
P68400	Q9UNN5
P78527	P04150
P78527	P04637
P78527	P05412
P78527	P07900
P78527	P11831
P78527	P15927
P78527	P49917
P78527	P78527
P98161	P04792
P98161	Q13671
P98161	Q15139
P98161	Q8WUI4
P98161	Q96A00
Q00526	P18846
Q00534	Q08999
Q00535	P12931
Q00535	P41236
Q00535	P49768
Q00535	Q02078
Q00535	Q15078
Q00535	Q15833
Q02156	P05783
Q02156	P08581
Q02156	P17302
Q02156	P18507
Q02156	P19429
Q02156	P46940
Q02156	P48751
Q02156	Q02156
Q02156	Q96A00
Q02156	Q9UBS0
Q02750	P27361
Q02750	P28482

Q02750	P49841
Q02763	Q02763
Q04912	Q04912
Q05397	P12814
Q05397	P49023
Q05397	Q05397
Q05397	Q99961
Q05513	O43315
Q05513	P31946
Q05513	P35568
Q05513	P35611
Q05513	P55040
Q05513	P55211
Q05513	Q04206
Q05513	Q05513
Q05513	Q96A00
Q05513	Q9UBS0
Q06187	P16885
Q06187	P42680
Q06187	P42768
Q06187	P51813
Q06187	P78347
Q06187	Q06187
Q06187	Q08881
Q07912	Q07912
Q08881	P10747
Q08881	P42680
Q08881	Q08881
Q08881	Q96LC7
Q12851	P02810
Q12866	Q12866
Q13043	P33778
Q13043	Q13043
Q13153	O95863
Q13153	P03372
Q13153	P04049
Q13153	P15056
Q13153	P16949
Q13153	P18669
Q13153	P19086
Q13153	P21333

Q13153	P36871
Q13153	P52565
Q13153	P53667
Q13153	P58012
Q13153	Q02750
Q13153	Q5VY93
Q13153	Q92934
Q13153	Q92974
Q13164	O00141
Q13164	P14921
Q13164	Q02078
Q13164	Q06413
Q13164	Q13163
Q13177	P01236
Q13177	P24844
Q13177	P35240
Q13233	O14733
Q13237	Q01970
Q13237	Q14847
Q13315	O14757
Q13315	O60934
Q13315	O75943
Q13315	O94916
Q13315	O96017
Q13315	P00519
Q13315	P04637
Q13315	P15336
Q13315	P15927
Q13315	P16104
Q13315	P16220
Q13315	P38398
Q13315	P49917
Q13315	P49959
Q13315	P54132
Q13315	P54274
Q13315	Q00987
Q13315	Q01094
Q13315	Q12888
Q13315	Q13315
Q13315	Q13541
Q13315	Q14191

Q13315	Q14683
Q13315	Q15831
Q13315	Q92878
Q13315	Q99638
Q13315	Q99708
Q13315	Q9BXW9
Q13315	Q9Y6K9
Q13418	P31749
Q13418	Q13418
Q13418	Q13765
Q13418	Q8TAE6
Q13418	Q96A00
Q13418	Q96C90
Q13464	O14974
Q13464	P08670
Q13464	P14136
Q13464	P15311
Q13464	P19105
Q13464	P35611
Q13464	P48436
Q13464	P60484
Q13464	Q05397
Q13523	P19419
Q13535	O14757
Q13535	P16220
Q13535	P38398
Q13535	Q00987
Q13535	Q01094
Q13535	Q8WXE1
Q13563	Q9BZL6
Q13627	O43597
Q13627	P37840
Q13627	P49418
Q13627	Q05193
Q13627	Q12778
Q13882	P49023
Q13882	Q13882
Q13976	O76074
Q13976	P35367
Q13976	P41220
Q13976	P52943

Q13976	P61224
Q13976	Q14847
Q13976	Q15637
Q13976	Q9Y613
Q13976	Q9Y6F6
Q14012	P05783
Q14012	Q14012
Q14012	Q16566
Q14164	Q04206
Q14164	Q14653
Q14289	Q14289
Q14680	P30305
Q15118	O00141
Q15118	O15530
Q15118	P05106
Q15118	P08559
Q15118	P23443
Q15118	P31749
Q15118	P31751
Q15118	P36507
Q15118	Q02156
Q15118	Q02750
Q15118	Q05513
Q15118	Q05655
Q15118	Q13153
Q15118	Q15418
Q15118	Q16512
Q15118	Q16513
Q15118	Q96BR1
Q15118	Q99683
Q15118	Q9HBY8
Q15118	Q9Y243
Q15119	O00141
Q15119	P08559
Q15120	P08559
Q15120	P31946
Q15120	Q04917
Q15208	Q15208
Q15349	P16220
Q15418	P17676
Q15418	P35568

Q15418	P50549
Q15418	Q15653
Q15418	Q16821
Q15418	Q92731
Q15418	Q92934
Q15569	P23528
Q15759	O60381
Q15759	P41235
Q15759	Q8IW41
Q15831	O60285
Q15831	P27448
Q15831	P57059
Q15831	P60484
Q15831	Q13131
Q15831	Q14680
Q15831	Q15831
Q15831	Q7KZI7
Q15831	Q8IWQ3
Q15831	Q8TDC3
Q15831	Q96L34
Q15831	Q9H093
Q15831	Q9H0K1
Q15831	Q9NRH2
Q15831	Q9P0L2
Q15831	Q9Y2K2
Q15835	P08100
Q16539	O75582
Q16539	O75676
Q16539	P03372
Q16539	P05787
Q16539	P14598
Q16539	P15336
Q16539	P17275
Q16539	P19419
Q16539	P19634
Q16539	P42574
Q16539	P49137
Q16539	Q02078
Q16539	Q06413
Q16539	Q07666
Q16539	Q14790

Q16539	Q14934
Q16539	Q15750
Q16539	Q8IW41
Q16539	Q8WYK2
Q16539	Q9NQU5
Q16539	Q9UBK2
Q16566	P16220
Q16566	P56524
Q16566	Q09472
Q16566	Q92793
Q16566	Q9UQL6
Q16584	P45985
Q16584	Q16584
Q16654	P08559
Q16659	P46695
Q16659	Q16659
Q2M2I8	P49757
Q2M2I8	Q96CW1
Q2M2I8	Q9BXS5
Q5S007	P26038
Q5VT25	P53667
Q5VT25	P53671
Q8IVT5	P04049
Q8IW41	P04637
Q8N5S9	Q8IU85
Q8TAS1	P02686
Q8TAS1	P17600
Q8TAS1	P46527
Q8TD19	Q8TD19
Q8TD19	Q8TDX7
Q8TD19	Q9HC98
Q8TDC3	P30305
Q8TDC3	P30307
Q8WZ42	O15273
Q92630	P04637
Q92630	Q14195
Q92918	Q13094
Q92918	Q16584
Q96GD4	O15392
Q96GD4	P08670
Q96GD4	P14136

Q96GD4	P17661
Q96GD4	P49450
Q96GD4	P68431
Q96GD4	P84243
Q96GD4	Q02241
Q96GD4	Q96GD4
Q96GD4	Q99661
Q96GD4	Q9H0H5
Q96GD4	Q9NQS7
Q96QT4	P35579
Q96RG2	Q96RG2
Q96S53	P23528
Q99558	Q00653
Q99683	P38936
Q99683	P45985
Q99683	Q99683
Q99986	P04637
Q99986	P05412
Q99986	P15336
Q99986	P68431
Q9BQI3	P05198
Q9BUB5	P06730
Q9H2X6	O00257
Q9H2X6	P04637
Q9H2X6	P29590
Q9H4A3	O95747
Q9H4A3	Q9UEW8
Q9H4B4	P30307
Q9HBH9	P06730
Q9HC98	O00141
Q9NWZ3	P51617
Q9NZJ5	P05198
Q9P286	Q9P286
Q9U5L0	P26678
Q9U5L0	P35270
Q9UBE8	Q9UJU2
Q9UBS0	P04792
Q9UBS0	P05783
Q9UHD2	Q04206
Q9UHD2	Q14653
Q9UIK4	Q9UIK4

Q9UKI8	Q99638
Q9UM73	P40763
Q9UM73	Q86WB0
Q9UM73	Q9UM73
Q9UPZ9	Q9UPZ9
Q9UQM7	P00533
Q9UQM7	P04626
Q9UQM7	P07101
Q9UQM7	P08670
Q9UQM7	P09917
Q9UQM7	P14921
Q9UQM7	P23677
Q9UQM7	P29475
Q9UQM7	P35367
Q9UQM7	P47712
Q9UQM7	P51790
Q9UQM7	Q00613
Q9UQM7	Q01970
Q9UQM7	Q92736
Q9UQM7	Q96A00
Q9UQM7	Q9UQM7
Q9Y2H1	Q9Y2H1
Q9Y463	O75533
Q9Y463	P20823
Q9Y463	P38936
Q9Y463	Q9Y463
Q9Y6E0	Q9Y2H1
